# Supplementary material for: Comorbidity, Radiation Duration, and Pretreatment Body Muscle Mass Predict Early Treatment Failure in Taiwanese Patients with Locally Advanced Oral Cavity Squamous Cell Carcinoma after Completion of Adjuvant Concurrent Chemoradiotherapy
Source: Diagnostics (Basel). 2021 Jul 2;11(7):1203. doi: 10.3390/diagnostics11071203 (PMC8306647; doi:10.3390/diagnostics11071203)
Supplement: Supplementary file 1 [file diagnostics-11-01203-s001.zip › Supplementary files/Table S1_DXA_LAOCSCC_Diagnostics.pdf]

Supplementary Table S1. Clinicopathologic features of 21 oral cavity cancer patients with early treatment failure

Case	Age	TNM stage, site	Hist. grade	PS	HN-CCI[b]	Smoking	Alcohol	Betel nut	LBMI[c]	ASMI[d]	Radiotherapy	Cisplatin dose[e]	Failure pattern	Time to treatment failure (months)
1	58	T4aN0M0, IVA tongue	3	2	0	No	No	No	13.7	4.99	62 Gy, 31 frx	240	PD	3.03
2	49	T4aN0M0, IVA mouth floor	3	2	0	Yes	Yes	Yes	13.9	5.88	66 Gy’ 33 frx	180	PD	2.50
3	65	T4aN0M0, IVA tongue	2	2	1	Yes	Yes	Yes	13.9	5.17	56 Gy, 28 frx	240	PD	4.57
4	54	T4aN0M0, IVA buccal mucosa	3	1	0	Yes	Yes	Yes	14.0	5.25	60 Gy, 30 frx	300	PD	5.97
5	39	T4aN2bM0, IVA buccal mucosa	2	2	0	Yes	Yes	Yes	12.1	5.53	62 Gy, 31 frx	240	PD	3.63
6	51	T4aN2cM0, IVA tongue	3	1	0	Yes	Yes	Yes	15.5	7.04	66 Gy, 33 frx	240	CD	0.87
7	61	T4aN2cM0, IVA buccal mucosa	2	1	1	Yes	Yes	Yes	15.0	6.09	62 Gy, 31 frx	160	NCD (sepsis)	3.57
8	61	T3N1M, III buccal mucosa	2	1	1	Yes	Yes	Yes	17.8	7.74	60 Gy, 30 frx	240	PD	3.73
9	49	T3N0M, III gingiva	3	1	0	Yes	Yes	No	14.8	5.45	64 Gy, 32 frx	240	CD	2.20
10	53	T4aN1M0, IVA buccal mucosa	2	1	0	Yes	Yes	Yes	13.3	5.37	64 Gy, 32 frx	240	CD	0.76
11	47	T4aN2cM0, IVA tongue	3	1	0	Yes	Yes	Yes	16.5	7.20	66 Gy, 33 frx	280	PD	4.83
12	67	T4aN2cM0, IVA buccal mucosa	3	1	1	No	Yes	No	15.8	6.02	66 Gy, 33 frx	240	PD	4.07
13	44	T4aN2cM0, IVA gingiva	3	1	0	Yes	Yes	Yes	16.5	7.33	64 Gy, 32 frx	240	PD	5.93
14	65	T4aN1M0, IVA tongue	2	2	1	Yes	Yes	Yes	13.8	5.37	64 Gy, 36 frx	280	PD	4.23
15	56	T4bN2bM0, IVB buccal mucosa	1	1	0	Yes	Yes	Yes	16.7	6.88	72 Gy, 36 frx	240	CD	1.03

16	56	T4bN2bM0, IVB buccal mucosa	3	1	0	Yes	Yes	Yes	15.9	7.09	66 Gy, 33 frx	240	PD	5.23
17	49	T4aN3M0, IVB buccal mucosa	3	0	0	Yes	Yes	Yes	15.3	6.75	72 Gy, 36 frx	240	PD	4.63
18	41	T3N2bM0, IVA buccal mucosa	3	1	1	Yes	Yes	Yes	16.9	8.01	66 Gy, 33 frx	240	PD	4.03
19	49	T3N3M0, IVB tongue	2	1	1	Yes	Yes	Yes	14.0	5.46	66 Gy, 33 frx	240	PD	3.27
20	59	T4aN1M0, IVA tongue	2	1	1	Yes	No	Yes	14.4	5.89	66 Gy, 33 frx	240	NCD (sepsis)	1.23
21	43	T4aN2aM0, IVA tongue	2	1	0	Yes	No	Yes	13.74	4.25	64 Gy, 32 frx	240	PD	5.20

PS, Eastern Cooperative Oncology Group performance status; HN-CCI, head and neck Charlson Comorbidity Index; LBMI, lean body mass index; ASMI, appendicular skeletal muscle index; frx, fraction; Time to treatment failure, the time period to develop treatment failure after CCRT ends; PD, disease in progression; CD, cancer death; NCD, non-cancer death

[a]Histological grade [b]0, no comorbidity; 1, having comorbidity [c]unit, kg/m^2 [d]unit, kg/m^2 [e]unit, mg/m^2
