# Supplementary material for: Comorbidity, Radiation Duration, and Pretreatment Body Muscle Mass Predict Early Treatment Failure in Taiwanese Patients with Locally Advanced Oral Cavity Squamous Cell Carcinoma after Completion of Adjuvant Concurrent Chemoradiotherapy
Source: Diagnostics (Basel). 2021 Jul 2;11(7):1203. doi: 10.3390/diagnostics11071203 (PMC8306647; doi:10.3390/diagnostics11071203)
Supplement: Supplementary file 1 [file diagnostics-11-01203-s001.zip › Supplementary files/Table S2_DXA_LAOCSCC in Factor 3_Diagnostics.pdf]

Table S2. The characteristic between patients with low Factor 3 (≤ 0.67) and high factor 3 (> 0.67) early treatment failure in 69 oral cavity cancer patients following CCRT completion

	Factor 3 level		
Variables, expressed as Numbers (%) or mean±SD	≤ 0.67	> 0.67	*P*-value
Patient number	52	17	
Clinicopathologic			
Age (years)	53.6 ± 7.9	51.8 ± 10.1	0.454
Tumor location			0.145
Buccal mucosa	16 (30.8)	4 (23.5)	
Tongue	23 (44.2)	5 (29.4)	
Gingiva	6 (11.5)	7 (41.2)	
Mouth floor	3 (5.8)	0 (0.0)	
Retromolar	2 (3.8)	0 (0.0)	
Lip	1 (1.9)	1 (5.9)	
Hard palate	1 (1.9)	0 (0.0)	
TNM stage (III *vs* IVA *vs* IVB)	2 (3.8) : 36 (69.2) : 14 (27.0)	2 (11.8) : 14 (82.3) : 1 (5.9)	0.117
T status (T0-2 *vs* T3-4)	5 (9.6) : 47 (90.4)	3 (17.9) : 14 (82.4)	0.368
N status (N0-1 *vs* N2-3)	22 (42.3) : 30 (57.7)	8 (47.1) : 9 (52.9)	0.732
ECOG performance status (0 : 1 : 2)	1 (19.1) : 45 (86.5) : 6 (11.6)	1 (5.9) : 16 (94.1) : 0 (0.0)	0.434
Histological grade (1 : 2 : 3)	8 (15.4) : 36 (69.2) : 8 (15.4)	0 (0.0) : 15 (88.2) : 2 (11.8)	0.187
Smoking (no : yes)	3 (5.8) : 49 (94.2)	3 (17.6) : 14 (82.4)	0.131
Alcohol (no : yes)	15 (28.8) : 37 (71.2)	3 (17.6) : 14 (82.4)	0.361
Betel nut (no : yes)	11 (21.2) : 41 (78.8)	5 (29.4) : 12 (70.6)	0.484
HN-CCI (0 *vs* 1 *vs* 2 *vs* ≥3)	23 (44.2):10 (19.2):3 (5.8):16 (30.8)	6 (35.3):5 (29.4):3 (17.6):3 (17.6)	0.353
Tracheostomy (no : yes)	15 (28.8) : 37 (71.2)	8 (47.1) : 9 (52.9)	0.167
Adjuvant CCRT due to risk factor			0.179
One major (positive surgical margin or ENE)	34 (65.4)	8 (47.1)	
≥ 3 Minors	18 (34.6)	9 (52.9)	
CCRT			
RT dose (Gy)	64.6 ± 3.8	63.5 ± 3.8	0.339
RT duration (days)	48.8 ± 5.2	45.8 ± 2.8	0.031*
Cisplatin dose (mg/m^2)	239.1± 20.7	236.4 ± 20.3	0.646
CCRT-induced grade 3/4 toxicity			
Dermatitis	3 (5.8)	0 (0.0)	0.311
Pharyngitis	5 (9.6)	0 (0.0)	0.194
Mucositis	13 (25.6)	3 (17.6)	0.680
Infection	10 (19.2)	0 (0.0)	0.043*

Emesis	6 (11.5)	0 (0.0)	0.194
Anemia (%)	3 (5.8)	2 (11.8)	0.408
Neutropenia (%)	21 (40.4)	2 (11.8)	0.022*
Thrombocytopenia (%)	2 (3.8)	2 (11.8)	0.225
Mean daily calorie intake during CCRT (kcal/kg/day)	30.0 ± 8.9	27.5 ± 10.6	0.221
Nutritional and inflammatory markers before CCRT			
BMI (kg/m^2)	21.5 ± 3.5	26.4 ± 4.5	<0.001*
BWL(kg)	-5.8 ± 8.6	15.1 ± 11.7	<0.001*
Hb (mg/dL)	11.7 ± 1.5	11.8 ± 1.4	0.603
WBC (x10^{3}cells/mm^3)	7.3 ± 2.7	7.1 ± 1.9	0.820
Platelet count (x10^3/mm^3)	347.5 ± 157.2	321.5 ± 119.0	0.535
Albumin (g/dL)	3.8 ± 0.5	4.0 ± 0.4	0.117
CRP (mg/dL)	12.4 ± 8.4	8.3 ± 9.8	0.037*
PG-SGA (well vs moderate vs severe) before CCRT	8 (15.4) : 29 (55.8) : 15 (28.8)	5 (29.4) : 9 (52.9) : 3 (17.6)	0.373
Body composition parameters			
Factor 1	-0.032 ± 0.98	0.099 ± 1.07	0.638
Factor 2	-0.017 ± 0.98	0.053 ± 1.02	0.803
Factor 3	-0.403 ± 0.77	1.233 ± 0.42	<0.001*
Factor 4	0.044 ± 1.07	-0.136 ± 0.75	0.522
Factor 5	-0.042 ± 1.03	0.127 ± 0.93	0.549

*indicates a significant *P*-value < 0.05

The analytic methods of Table S2 are identical to those of Table 3

Abbreviations: CCRT, concurrent chemoradiotherapy; TNM, tumor node metastasis; ECOG, Eastern Collaboration Oncology Group; HN-CCI, head and neck Charlson Comorbidity Index; RT, radiotherapy; PG-SGA, patient-generated subjective global assessment; BMI, body mass index; BWL, body weight loss; Hb, hemoglobin; WBC, white cell count; TLC, total lymphocyte count; CRP, C-reactive protein

Independent *t* test was used for age, BMI, Hb, platelet count and Albumin. Mann-Whitney test was used for BWL, WBC, CRP and all body composition parameters
