# Supplementary material for: Comorbidity, Radiation Duration, and Pretreatment Body Muscle Mass Predict Early Treatment Failure in Taiwanese Patients with Locally Advanced Oral Cavity Squamous Cell Carcinoma after Completion of Adjuvant Concurrent Chemoradiotherapy
Source: Diagnostics (Basel). 2021 Jul 2;11(7):1203. doi: 10.3390/diagnostics11071203 (PMC8306647; doi:10.3390/diagnostics11071203)
Supplement: Supplementary file 1 [file diagnostics-11-01203-s001.zip › Supplementary files/Table S3_DXA_LAOCSCC in icomplete CCRT and data.pdf]

**Table S3.** The characteristic between patients with complete CCRT and incomplete CCRT/data collection in 85 oral cavity cancer patients following curative surgery

| | CCRT completion | | |
|---|---|---|---|
| Variables, expressed as Numbers (%) or mean±SD | Yes | No | *P*-value |
| ***Patient number*** | 69 (100) | 16 (100.0) | |
| ***Clinicopathologic*** | | | |
| Age (years) | 53.2 ± 8.4 | 57.69 ± 10.9 | 0.077 |
| Tumor location | | | 0.812 |
| Buccal mucosa | 20 (29.0) | 4 (25.0) | |
| Tongue | 28 (40.6) | 7 (43.8) | |
| Gingiva | 13 (18.8) | 2 (12.5) | |
| Mouth floor | 3 (4.3) | 2 (12.5) | |
| Retromolar | 2 (2.9) | 0 (0.0) | |
| Lip | 2 (2.9) | 1 (6.3) | |
| Hard palate | 1 (1.4) | 0 (0.0) | |
| TNM stage (III *vs* IVA *vs* IVB) | 4 (5.8) : 50 (72.5) : 15 (21.7) | 2 (12.5) : 8 (50.0) : 6 (37.5) | 0.213 |
| T status (T0-2 *vs* T3-4) | 19 (27.5) : 50 (72.5) | 2 (12.5) : 14 (87.5) | 0.919 |
| N status (N0-1 *vs* N2-3) | 30 (43.4) : 39 (46.6) | 3 (31.2) : 13 (68.8) | 0.371 |
| ECOG performance status (0 : 1 : 2) | 2 (2.9) : 61 (88.4) : 6 (8.6) | 1 (6.2) : 13 (81.3) : 2 (12.5) | 0.448 |
| Histological grade (1 : 2 : 3) | 8 (11.6) : 51 (73.9) : 10 (14.5) | 2 (12.5) : 12 (75.0) : 2 (12.5) | 0.976 |
| Smoking (no : yes) | 6 (8.7) : 63 (91.3) | 0 (0.0) : 16 (100.0) | 0.221 |
| Alcohol (no : yes) | 18 (26.1) : 51 (73.9) | 3 (18.8) : 13 (81.2) | 0.540 |
| Betel nut (no : yes) | 16 (23.2) : 53 (76.8) | 8 (50.0) : 8 (50.0) | 0.032* |
| HN-CCI (0 *vs* 1 *vs* 2 *vs* ≥3) | 29 (42.0): 15 (21.7): 6 (8.7): 19 (27.5) | 4 (25.0):8 (50.0):3 (18.3):1 (6.3) | 0.049* |
| Tracheostomy (no : yes) | 23 (33.4) : 46 (66.6) | 9 (56.3) : 7 (43.8) | 0.088 |
| Adjuvant CCRT due to risk factor | | | 0.089 |
| One major (positive surgical margin or ENE) | 42 (60.9) | 6 (37.5) | |
| ≥ 3 Minors | 27 (39.1) | 10 (62.5) | |
| ***Nutritional and inflammatory markers before CCRT*** | | | |
| BW (kg) | 63.6 ± 12.6 | 60.7 ± 10.5 | 0.407 |
| BMI (kg/m$^2$) | 22.8 ± 4.3 | 22.4 ± 3.5 | 0.793 |
| BWL(kg) | 1.0 ± 6.3 | 0.92 ± 11.7 | 0.885 |
| Hb (mg/dL) | 11.7 ± 1.5 | 11.6 ± 1.7 | 0.884 |
| WBC (x10$^3$cells/mm$^3$) | 7.3 ± 2.5 | 7.2 ± 4.6 | 0.979 |
| Platelet count (x10$^3$/mm$^3$) | 341.1 ± 148.4 | 224.8 ± 82.2 | 0.003* |
| Albumin (g/dL) | 3.9 ± 0.5 | 3.8 ± 0.6 | 0.848 |
| CRP (mg/dL) | 9.3 ± 13.7 | 13.2 ± 14.4 | 0.670 |
| ***PG-SGA (well vs moderate vs severe) before CCRT*** | 2 (2.9) : 32 (46.4) : 35 (50.7) | 2 (12.5) : 10 (62.5) : 4 (25.0) | 0.807 |

| | | | |
|---|---|---|---|
| ***Body composition parameters before CCRT*** | | | |
| LBMI (kg/m$^2$) | 15.7 ± 1.6 | 16.3 ± 2.4 | 0.212 |
| TFMI (kg/m$^2$) | 6.1 ± 3.1 | 9.2 ± 3.2 | 0.001* |
| ASMI (kg/m$^2$) | 6.6 ± 1.0 | 6.7 ± 1.1 | 0.666 |
| Android (%) | 29.7 ± 13.4 | 25.4 ± 10.4 | 0.239 |
| Gynoid (%) | 25.7 ± 8.3 | 24.3 ± 8.2 | 0.514 |
| BMCI (kg/m$^2$) | 1.4 ± 0.1 | 1.4 ± 1.1 | 0.881 |

*indicates a significant *P*-value < 0.05

The analytic methods of Table S3 are identical to those of Table 3

Abbreviations: CCRT, concurrent chemoradiotherapy; TNM, tumor node metastasis; ECOG, Eastern Collaboration Oncology Group; HN-CCI, head and neck Charlson Comorbidity Index; RT, radiotherapy; PG-SGA, patient-generated subjective global assessment; BMI, body mass index; BWL, body weight loss; Hb, hemoglobin; WBC, white cell count; TLC, total lymphocyte count; CRP, C-reactive protein

Independent *t* test was used for age, BMI, Hb, platelet count and Albumin. Mann-Whitney test was used for BWL, WBC, CRP and all body composition parameters
